# Cinematic rendering for three-dimensional reconstructions of the chest wall: a new reality

**DOI:** 10.31744/einstein_journal/2020MD5223

**Published:** 2020-01-31

**Authors:** Altair da Silva Costa, Norman Gellada

**Affiliations:** 1 Universidade Federal de São Paulo São PauloSP Brazil Universidade Federal de São Paulo, São Paulo, SP, Brazil.; 2 Cedars-Sinai S. Mark Taper Foundation Imaging Center Los AngelesCA United States Cedars-Sinai S. Mark Taper Foundation Imaging Center, Los Angeles, CA, United States.

**Keywords:** Tomography, x-ray computed, Image processing, computer-assisted, Image processing, computer-assisted

## Abstract

Computed tomography with multiple detectors and the advancement of processors improved rendered images and three-dimensional reconstructions in clinical practice. Traditional axial slices form non-intuitive images because they are seen in only one plane. The three-dimensional reconstructions can show structures details and diseases with complex anatomy in different perspectives. Cinematic rendering is a newly three-dimensional reconstruction technique, already approved for clinical use, which can produce realistic images from traditional computed tomography data. The algorithm used is based on light trajectory methods and the global lighting model, which simulate thousands of images from all possible directions. Thus, the technique shapes the physical propagation of light and generates a realistic three-dimensional image with depth, shadows and more anatomic details. It is a multidimensional rendering acquired through complex lighting effects. The aim of this article was to show the advance of three-dimensional technology with the cinematic rendering in images exams of the thoracic wall.

## INTRODUCTION

Multidetector computed tomography (CT) has changed medical imaging. Multiple rows of detector allow faster image acquisition with higher resolution. Along with equipment, novel two or three-dimensional post-processing techniques have been developed, providing excellent image quality.^1,2^ Multiplanar (axial, sagittal or coronal) two-dimensional techniques with sub-millimetric resolution are routinely used in radiology practice. Three-dimensional image post-processing and volume rendering techniques are used primarily for clear, objective depiction of complex anatomical changes.^3^

Three-dimensional reconstructions of conventional helical CT images are widely available. Still, greater resolution provided by multiple detectors and more advanced processors have led to greater use of three-dimensional rendering in clinical practice.^1,2^

Three-dimensional reconstructions provide all-encompassing images depicting non-intuitive anatomical relations compared to traditional axial images. Volume rendering techniques play an increasingly significant role in medical imaging.^1,2^ Three-dimensional visualization of CT image data may provide relevant information compared to two-dimensional axial images, both for surgical planning and postoperative follow-up.^4^

At first, volume rendered images required considerable post-processing time and user intervention due to the limited capacity of computers to process high volume data. Powerful computers are now capable of near real-time processing and automatic rendering for three-dimensional visualization.^1-3^ The possibility of simultaneous visualization of different anatomical structures of a larger body region in colors introduced by volume rendering has changed imaging assessment standards. This different depiction of image data may be useful for anatomically complex structures and diseases and provides a more user-friendly illustration of imaging findings for medical training and treatment planning.^1,2^

### Volume rendering techniques

In medical radiology, tissue information transcribed via magnetic resonance signal intensity, sonographic echogenicity and CT attenuation is transformed into encoded data. Data are then processed using mathematical algorithms to generate images in shades of gray (Hounsfield units) or encoded in colors.^1,2^ In two-dimensional techniques, such as maximum and minimum intensity projections, a single volumetric parameter from the original data is used for image reconstruction. However, more complex algorithms can be used to create three-dimensional reconstructions for improved visualization of complex structures and anatomical relations. Volume rendering has been the most widely used three-dimensional reconstruction technique in clinical practice to date.^1,2,5^

Volume rendering also allows real-time manipulation and customization of three-dimensional images to depict anatomical details from different perspectives. The technique is based on ray casting and local illumination modelling principles stating that for each pixel on a screen one ray is cast through the volume and intersects a line of voxels. Volume rendering can easily be computed using a Riemann integral, but neglects complex light ray paths, such as scattering and light extinction, yielding more artificial looking images.^1,2^ As a light ray passes through the volume, specific steps must be applied by the algorithm before the final image is formed, such as sampling, classification and compositing. All volume rendering algorithms use this process. As a light ray passes through the volume, volume contributions are accumulated and calculated at specific points along the ray using the aforementioned sampling process. Each sample point is classified and assigned a color and an opacity value via transfer functions. This step allows the construction of a color representation from gray scale sections. Composition is the process by which color and opacity values from each line of sample points are accumulated using a mathematical formula to generate a final projection at the corresponding pixel.^1,2^ Each voxel contains one or more types of tissue. Each tissue is determined and represented by a percentage number ranging from zero to 100 according to a predefined attenuation threshold level, and is assigned color and opacity values. The weighted sum of these values for tissues in a voxel is then calculated to determine color and transparency. This step is repeated for each voxel sampled along the light ray passing through the volume. The values of all points sampled along the ray are accumulated and projected to form the final image. The software analyses different combinations of Hounsfield unit ranges to show different types of tissues and respective locations, then generates a three-dimensional image based on observer and light source positions.^1,2^

The advantage of volume-rendered over classical CT reconstructions is the analysis of comprehensive data contained in volume-rendered images, creating an accurate three-dimensional representation of target tissues. This allows visualization of larger volumes in isolated images and simultaneous assessment of distant structures and their spatial relations.^1,2^

## CINEMATIC RENDERING

**Figure 1:**
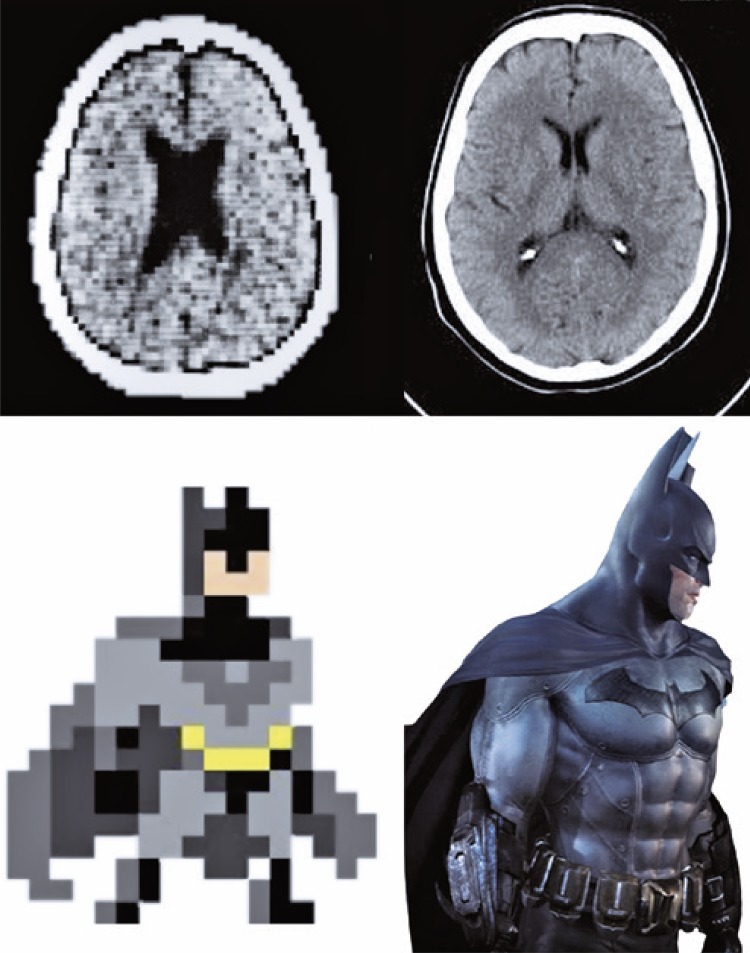
Head tomography and digital drawing of Batman, from 1987 and 2017

Cinematic rendering is a recent three-dimensional reconstruction technique. Cinematic rendering can generate realistic three-dimensional images from traditional CT and magnetic resonance data and has been approved for clinical. The technique was inspired by the success enjoyed by the computer-animated movie industry, Pixar Animation Studios in particular, in creating highly realistic characters (hence the name cinematic rendering).^1,2^ Differences in image resolution can be appreciated in tomographic images and images derived from the games industry over a 30-year period (1987 to 2017) (Figure 1).

Overall, cinematic rendering uses an algorithm based on path of light ray and global illumination models that simulate the different paths of billions of photons instead of ray casting methods, in which each pixel is formed by one ray of light. The software simulates millions of images in all possible directions within a volumetric dataset and their interactions to form one voxel.^1,2^ The technique models the physical propagation of light to generate a realistic three-dimensional image based on acquired data, and is therefore a multidimensional rendering acquired via rays of light. Given light ray paths may be (in theory) be infinite, simulations of the Monte Carlo equation are used to generate a random subset of light paths with adequate distribution.^1,2^ Such high complexity requires the use of importance sampling (a parallelization and optimization algorithm), modeling of scattering and reflection. The final processed image is obtained by the progressive mean of several simulations of radiance from illumination effects in random directions. To attain a realistic quality, high dynamic range rendering light maps are used to create a natural lighting environment.

Using this technical process, complex lighting effects are obtained, such as soft shadows, depth of field, surface scattering, refraction, absorption and environment variations.^1,2^

Useful as they may be for complex anatomy assessment, volume rendering techniques are not perfect and have some downsides, such as potential masking of anatomical information and pathological changes. Therefore, images must be interpreted by trained, experienced professionals.^2^ Visualization tools (*i.e.*, filtering and subtraction) increase diagnostic accuracy. The fact that nothing is added to what is actually present in original images acquired from patients must be emphasized. In dubious cases, reconstructions must be correlated and compared with corresponding original multiplane images.^2^

This paper set out to describe advancements in three-dimensional cinematic volume rendering technology in chest wall imaging.

This project was approved by the Ethics Committee of *Universidade Federal de São Paulo* (UNIFESP), opinion number 3.006.423, CAAE: 96263018.6.0000.5505.

Images were processed using syngo.via^®^ software (Siemens). Isotropic images were downloaded to the MMreading (multimodal) protocol. Window level adjustments for depiction of the target anatomical region were applied to volume rendered images. Axial, coronal and sagittal images were converted to cinematic images using the rendering algorithm.

**Figure 2:**
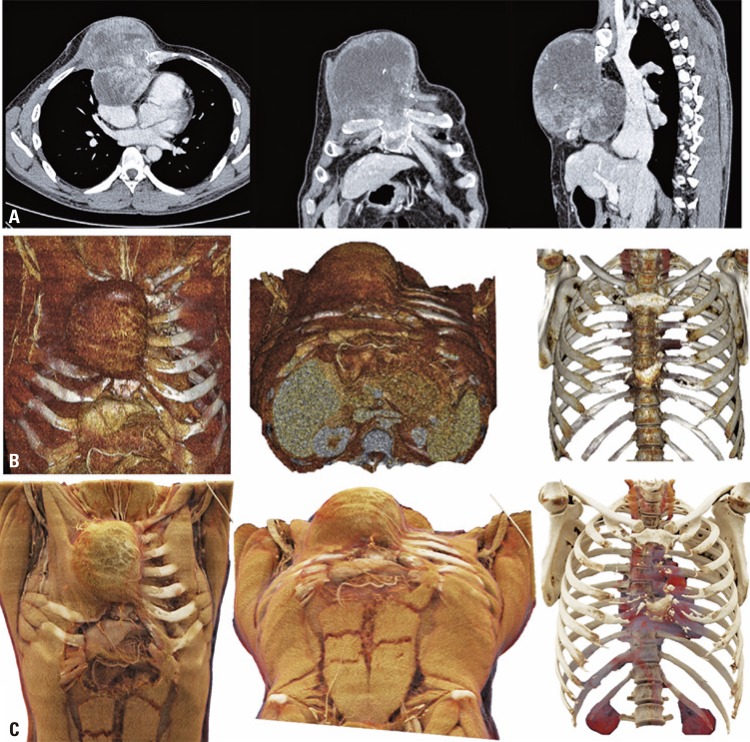
Synovial sarcoma of the chest wall. A) Conventional tomography; B) Volume rendering; and C) Cinematographic rendering

**Figure 3:**
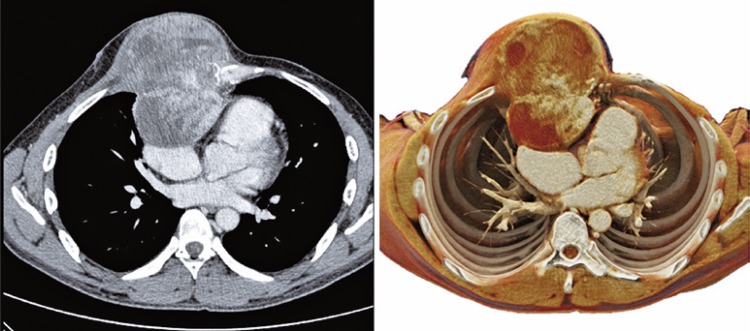
Synovial sarcoma of the chest wall - axial section, conventional tomography and cinematographic rendering

The first example is of a 34-year-old male patient, presenting with synovial sarcoma metastasis to the anterior chest wall (Figures 2 and 3).

**Figure 4:**
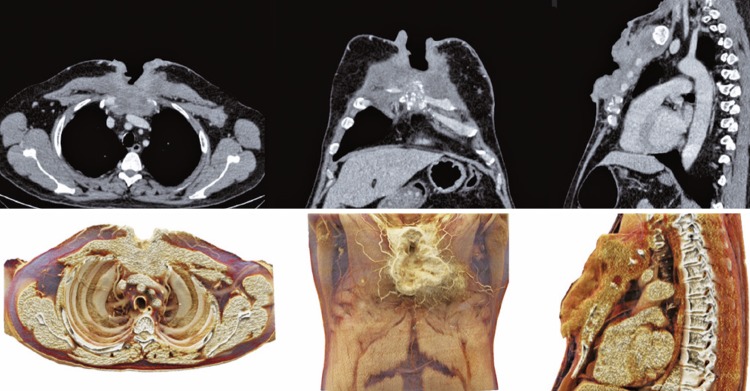
Chest wall carcinoma. Axial, coronal and sagittal sections - conventional tomography and cinematographic volume rendering

**Figure 5 f05:**
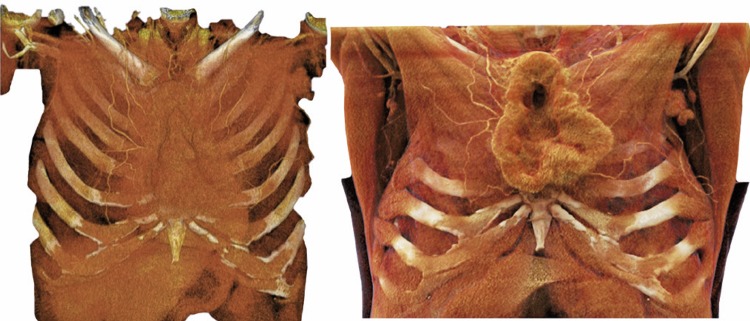
Chest wall carcinoma. Coronal section comparison of conventional tomography and cinematographic rendering

**Figure 6 f06:**
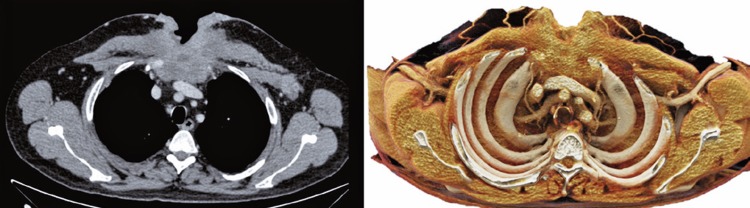
Chest wall carcinoma. Axial section comparison of conventional tomography and cinematographic rendering

The second example is of a 53-year-old male patient presenting with squamous-cell carcinoma on the anterior chest wall (Figures 4 to 6 [Fig f05] [Fig f06]).

## DISCUSSION

Volume and cinematic rendering share the same general concepts: data segmentation based on voxel attenuation and conversion of opacity and brightness to colors. High brightness structures, such as contrast-filled vessels and bones can be depicted with high image quality, whereas low to moderate brightness structures, such as soft tissues, are depicted with low image quality. Cinematic rendering provides more realistic images of anatomy and disease, including low brightness structures.^2^In both techniques, the brightness of each voxel is defined by the distribution of these light properties. In volume rendering, differences in light emitted to voxels are small. In contrast, cinematic rendering uses a more complex lighting model, with illumination effects for other voxels and subsequent reflections. The system also calculates the effect of body parts blocking the light to other structures and casting shadows in images, resulting in a more realistic perception of depth.^1,2^ The more complex lighting model involved in cinematic CT demands higher computing power. Hence, real-time display and three-dimensional manipulation (*e.g*., image rotation) are currently interrupted by repetitive calculation procedures. Accurate rendering of final images takes 5 to 30 seconds, depending on resulting image quality.^1,2^

Processing and segmentation techniques with three-dimensional reconstruction of medical images allow the construction of real anatomical models based on patient data. Therefore, three-dimensional images are true, authentic models of disease.^4,6^ To obtain such models, thin-slice CT (less than 3mm) providing adequate image quality is required. Computer-aided design software require a high number of images (more than 200 slices in DICOM format) for segmentation and generation of a good-quality three-dimensional polygon mesh.^1,4^ Examinations with less than 100 images or with 10mm slices generate excessive interference, yielding gross, poor quality images.^1,2,4^

Virtual or printed reconstructed three-dimensional models allow the reproduction of sophisticated anatomical structures and can be used to facilitate anatomical studies, surgical planning and development of novel techniques or devices. We are witnessing a revolution.^4-7^

### Potential opportunities

Three-dimensional technology may be applied in three major medical fields: teaching of undergraduates and residents, surgical planning and simulation, and patient education.^4-6^

### Anatomy teaching

Medical imaging has become an increasingly important tool in anatomy teaching. Three-dimensional technology has the potential to improve virtual anatomy even further and adds value by providing more realistic, patient-based images^4,6,8^ of normal as well as altered anatomy associated with several diseases. Students may interact with the anatomy of different patients and diseases anytime via a three-dimensional technology workstation.^6,8^

### Patient education

Good image quality provided by three-dimensional technology may improve communication with patients. “One image is worth a thousand words.” Physicians and surgeons can use three-dimensional images to illustrate disease features and potential treatments. Three-dimensional images are much better than traditional flat and cross-sectional images in shades of gray for patient education purposes, as the latter are seldom conducive to intuitive interpretation.^4,6,8^ Three-dimensional images are also unique, customized, faithful representations of true organs and diseases. Three-dimensional images may contribute more to patient understanding of disease and commitment to treatment plans compared to traditional medical images.^3,7^

### Surgical planning

Surgical planning and preoperative simulation are additional advantages of three-dimensional imaging. Three-dimensional image- and model-based planning have revolutionized surgical practice.^3-5,7^ A growing number of medical specialties are incorporating these technological resources into routine practice.^5,7^

Surgical planning involves mental integration of multiple images and is highly dependent on surgeon’s expertise. Newly-graduated physicians tend to have difficulties grasping three-dimensional anatomical changes displayed in traditional CT images.^4-8^ In contrast, three-dimensional technology provides a realistic representation of target structures in a single image. Also, image processing allows visualization of anatomical details from different perspectives. More realistic images may mirror what physicians see during surgery and procedures more faithfully compared to traditional (cross-sectional or flat) images, potentially mitigating the impact of incidental findings and anatomical variations.^4,6,7^ The potential of cinematic reconstruction for delineation of tumor limits, surgical planning and patient education has been emphasized in a study with pelvic tumors.^9^ In a different study involving 18 surgeons and comparing the value of cinematic rendering and conventional CT for the understanding of tomographic surgical anatomy in 40 patients, cinematic rendering allowed faster and more accurate understanding of surgical anatomy compared to conventional CT, regardless of surgeon expertise.^10^

### Limitations and alternative strategies

Current challenges associated with three-dimensional technology include original image quality, cost, time and structure.^2,3,5,7^ Investments in three-dimensional anatomy laboratories require equipment, software and trained support personnel.^1-3^

This novel technique has the potential to introduce a paradigm shift in virtual anatomy visualization. New image standards in medical reports should account for relevant changes detected in imaging assessments based on three-dimensional volume or cinematic reconstruction. It no longer makes sense to provide 200 to 300 conventional tomographic images instead of some good quality reconstructions of an affected organ. Differences in cost, storage and quality of three-dimensional compared to traditional axial images obtained via conventional CT should be accounted for.

Future studies are warranted to determine the potential applicability of cinematic rendering compared to conventional imaging or volume rendering.

## CONCLUSION

Three-dimensional reconstruction techniques such as volume rendering play an increasingly significant role in different medical specialties. Cinematic rendering is yet another three-dimensional reconstruction technique providing a more realistic view of anatomical structures, with more accurate shape and depth perception. These techniques are widely applicable to medical education, surgical planning and disease perception by patients.
