# Supplementary material for: Cinematic rendering for three-dimensional reconstructions of the chest wall: a new reality
Source: Einstein (Sao Paulo). 2020 Jan 31;18:eMD5223. doi: 10.31744/einstein_journal/2020MD5223 (PMC6999188; doi:10.31744/einstein_journal/2020MD5223)
Supplement: Translation

# Translation: Uma nova realidade: renderização cinematográfica para reconstruções tridimensionais da parede torácica

**Authors:** Altair da Silva Costa, Norman Gellada

**Affiliations:** 1 Brasil Universidade Federal de São Paulo, São Paulo, SP, Brasil.; 2 United States Cedars-Sinai S. Mark Taper Foundation Imaging Center, Los Angeles, CA, United States.

**Keywords:** Tomografia computadorizada por raios x, Processamento de imagem assistida por computador, Imagem tridimensional

## Abstract

A tomografia computadorizada com os múltiplos detectores e o avanço dos processadores melhoraram as imagens renderizadas e as reconstruções tridimensionais na prática clínica. Os cortes axiais tradicionais formam imagens não intuitivas, pois são vistas em apenas um plano. Já as reconstruções tridimensionais podem exibir detalhes anatômicos em diferentes perspectivas das estruturas e de doenças com anatomia complexa. A renderização cinematográfica é uma técnica de reconstrução tridimensional recentemente introduzida, já aprovada para uso clínico, que pode produzir imagens realistas a partir de dados tradicionais da tomografia computadorizada. O algoritmo usado é baseado em métodos de trajetória da luz e no modelo de iluminação global, os quais simulam milhares de imagens de todas as direções possíveis. Assim, a técnica molda a propagação física da luz e gera uma imagem tridimensional realista, com profundidade, sombras e mais detalhes da alteração anatômica. É uma renderização multidimensional adquirida por efeitos de iluminação complexos. O objetivo deste artigo foi mostrar o avanço da tecnologia tridimensional com a renderização cinematográfica nos dos exames de imagens da parede torácica.

## INTRODUÇÃO

A tomografia computadorizada (TC) com múltiplos detectores mudou a imagem médica. As múltiplas fileiras de detectores permitem aquisições mais rápidas e com maior resolução. Além do equipamento, houve um avanço importante nos programas com técnicas de pós-processamento em duas e três dimensões, com excelente qualidade de imagem.^1,2^ As técnicas bidimensionais multiplanares (axial, sagital ou coronal) são parte da rotina radiológica diária, com resolução submilimétrica. As técnicas de pós-processamento de imagens tridimensionais e de renderização por volume são usadas principalmente para demostrar, de maneira clara e objetiva, a complexidade da anatomia alterada.^3^

Embora as reconstruções tridimensionais já estivessem disponíveis com TC helicoidal convencional, a maior resolução alcançada com os múltiplos detectores e o avanço dos processadores melhoraram a utilização de imagens renderizadas tridimensionais na prática clínica.^1,2^

Reconstruções tridimensionais formam uma imagem abrangente, com visualização de relações anatômicas não tão intuitivas quando vistas apenas em cortes axiais tradicionais. As técnicas de renderização de volume desempenham papel cada vez mais importante nas práticas de imagens médicas.^1,2^ A visualização tridimensional dos dados da imagem da TC pode oferecer informações importantes em comparação com as imagens axiais bidimensionais, tanto para o planejamento pré-operatório quanto para o acompanhamento pós-tratamento.^4^

As primeiras gerações de imagens de volume eram associadas a longos períodos pós-processamento, com considerável necessidade de intervenção do usuário. Era nítida a limitação da capacidade do computador em executar o alto volume de dados. Atualmente, os poderosos computadores permitem processamento em tempo quase real e renderização automática para a visualização tridimensional.^1-3^ A renderização por volume mudou o padrão de avaliação dos exames de imagem, pois possibilita visualizar diferentes estruturas anatômicas de uma região do corpo maior ao mesmo tempo e em cores. Ela acrescenta uma representação diferente dos dados de imagem, que podem ser úteis para estruturas e doenças anatomicamente complexas. Ainda, proporciona uma ilustração mais fácil e compreensível dos achados de imagem para os médicos, sendo útil no ensino e no planejamento do tratamento.^1,2^

### Técnica de renderização de volume

Na radiologia médica, a informação tecidual, transcrita pela intensidade do sinal de ressonância magnética, pela ecogenicidade do ultrassom e pela atenuação da TC, é transformada em dados codificados. Os dados são processados por algoritmos matemáticos para gerar imagens em escala de cinza (unidades Hounsfield) ou codificadas por cores.^1,2^ Em técnicas bidimensionais, como projeção de intensidades máxima e de mínima, um único parâmetro volumétrico dos dados originais é usado para reconstruir imagens. Algoritmos mais complexos, no entanto, podem ser usados para gerar reconstruções tridimensionais que melhorem a visualização de estruturas complexas e relações anatômicas. Até o momento, a renderização volumétrica tem sido a técnica mais utilizada para reconstruções tridimensionais na prática clínica.^1,2,5^

A renderização volumétrica também possibilita que uma imagem tridimensional seja manipulada e personalizada em tempo real para exibir detalhes anatômicos de diferentes perspectivas. Baseia-se nos princípios de modelagem de raio e iluminação local, que afirmam que, para cada *pixel* de uma tela, um raio é lançado através do volume e cruza uma linha de *voxels*. Essa técnica é simples de ser computada com uma integral de Riemann, mas negligencia caminhos de luz complexos, como dispersão e extinção de luz, resultando em imagens com aparência mais artificial.^1,2^ À medida que o raio de luz passa pelo volume, etapas específicas devem ser aplicadas pelo algoritmo, antes que a imagem final seja formada. Essas etapas incluem amostragem, classificação e composição. Todos os algoritmos de renderização de volume utilizam esse processo. Conforme um raio de luz passa pelo volume, as contribuições de volume são acumuladas e calculadas em pontos específicos ao longo deste raio pelo processo de amostragem. Cada ponto de amostragem é classificado e recebe uma cor e um valor de opacidade via funções de transferência. Este passo permite a formação de uma representação de cor a partir de seções em escala de cinza. Composição é o processo pelo qual os valores de cor e opacidade de cada linha de pontos de amostra são acumulados por meio de fórmula matemática para gerar a projeção final no *pixel* correspondente.^1,2^ Cada *voxel* contém um ou mais tipos de tecido. Cada tecido é determinado e representado por um número percentual, que varia de zero a 100, com base em um nível de limiar de atenuação predefinido, sendo atribuídos valores de cor e opacidade. A soma ponderada desses valores para tecidos em um *voxel* é então calculada para determinar a cor e a transparência. Essa etapa é repetida para cada *voxel* amostrado ao longo do raio de luz que passa pelo volume. Os valores de todos os pontos amostrados ao longo de um raio são acumulados e projetados para contribuir para a imagem final. O software analisa as diferentes combinações de faixas de unidades Hounsfield para mostrar diferentes tipos de tecidos e sua localização e, em seguida, gera uma imagem tridimensional com base nas posições do observador e da fonte de luz.^1,2^

A vantagem do volume renderizado sobre as reconstruções clássicas de TC é a análise de dados abrangente das imagens renderizadas por volume, que cria uma representação tridimensional precisa dos tecidos-alvo. Isso permite a visualização de volumes maiores em imagens isoladas, e avaliações contemporâneas de estruturas distantes e suas relações espaciais.^1,2^

## RENDERIZAÇÃO CINEMATOGRÁFICA

A renderização cinematográfica é uma técnica de reconstrução tridimensional recentemente introduzida, já aprovada para uso clínico, que pode produzir imagens tridimensionais realistas, a partir de dados tradicionais de TC e ressonância magnética. Essa técnica foi inspirada pelo sucesso da indústria de animação, especialmente a *Pixar Animation Studios*, na criação de personagens de aparência altamente realista − daí o nome de renderização cinematográfica.^1,2^ Podemos observar a diferença na evolução da resolução por meio da evolução das imagens tomográfica e da indústria de jogos em 30 anos (1987 a 2017) (Figura 1).

**Figura 1:** Tomografia de crânio e desenho digital do Batman, de 1987 e 2017

No geral, para a renderização cinematográfica, ao invés de métodos de fundição de raios, em que cada *pixel* é formado por um raio de luz, o algoritmo usado é baseado em métodos de trajetória e no modelo de iluminação global, que simulam os diferentes caminhos de bilhões de fótons. O programa simula milhares de imagens, em todas as direções possíveis de um conjunto de dados volumétricos e suas interações para formar um *voxel*.^1,2^ Assim, a técnica molda a propagação física da luz e gera uma imagem tridimensional realista, com base nos dados obtidos. É uma renderização multidimensional adquirida por meio dos raios de luz. Como os caminhos de luz que podem ser traçados são, em teoria, infinitos, as simulações da equação de Monte Carlo são usadas para gerar um subconjunto aleatório de passagem de luz com distribuição adequada.^1,2^ Devido à tamanha complexidade, ainda se faz necessário usar a amostragem de importância (um algoritmo de paralelização e otimização), a modelagem de espalhamento e a reflexão. A imagem final processada é obtida pela média progressiva de numerosas simulações da radiância dos efeitos de iluminação em direções aleatórias. Para atingir a qualidade realista, mapas de luz de renderização de alta faixa dinâmica são utilizados para criar um ambiente de iluminação natural. Por este processo técnico, são obtidos efeitos de iluminação complexos, como sombras suaves, profundidade de campo, espalhamento superficial, refração, absorção e variação de ambiente.^1,2^

A técnica de renderização de volume não é perfeita e apresenta algumas desvantagens. Embora seja útil na avaliação da anatomia complexa, ela também pode mascarar informações anatômicas e algumas alterações patológicas. Por isso, é importante a presença de um profissional treinado e capacitado para interpretar as imagens.^2^ As ferramentas de visualização, os filtros e as subtrações auxiliam na precisão diagnóstica. Cabe ressaltar que o processo nunca adiciona informações ao leitor além do que está presente nas imagens originais adquiridas do paciente. Em caso de dúvida, as reconstruções sempre devem ser correlacionadas e comparadas com as imagens multiplanares originais correspondentes.^2^

O objetivo deste artigo foi mostrar o avanço da tecnologia tridimensional com a renderização cinematográfica nos dos exames de imagens da parede torácica.

Este projeto foi aprovado pelo comitê de ética da Universidade Federal de São Paulo (UNIFESP) parecer 3.006.423, CAAE: 96263018.6.0000.5505.

As imagens foram processadas pelo programa syngo.via^®^ (Siemens). As imagens isotrópicas foram carregadas no protocolo MMreading (multimodalidade). Os ajustes no nível da janela para exibir a área anatômica específica de interesse foram aplicados à imagem renderizada por volume. Com o algoritmo de renderização, as imagens foram convertidas para cinematográfica, nos planos axial, coronal e sagital.

O primeiro exemplo é o de um paciente do sexo masculino, 34 anos, com metástase de sarcoma sinovial para parede torácica anterior (Figuras 2 e 3).

**Figura 2:** Sarcoma sinovial parede torácica. A) Tomografia convencional; B) Renderização por volume; e C) Renderização cinematográfica

**Figura 3:** Sarcoma sinovial parede torácica − corte axial, tomografia convencional e renderização cinematográfica

O segundo exemplo é o de um paciente do sexo masculino, 53 anos, com carcinoma espino-celular em parede torácica anterior (Figuras 4 a 6 [Fig f05002] [Fig f06002]).

**Figura 4:** Carcinoma parede torácica. Cortes axial, coronal e sagital - tomografia convencional e renderização por volume cinematoráfica

**Figura 5 f05002:** Carcinoma parede torácica. Comparação corte coronal da tomografia convencional e renderização cinematoráfica

**Figura 6 f06002:** Carcinoma parede torácica. Comparação corte axial da tomografia convencional e renderização cinematoráfica

## DISCUSSÃO

As renderizações por volume e cinematográfica têm os mesmos conceitos gerais em comum: a segmentação de dados com base na atenuação de *voxels* e a conversão da opacidade e o brilho em cores. As estruturas de alto brilho, como vasos com contraste e ossos, podem ser representadas com qualidade elevada; já as estruturas com brilho moderado e baixo, como tecidos moles, a qualidade fica baixa. A renderização cinematográfica proporciona imagens mais realistas da anatomia e da doença, inclusive em estruturas com brilho baixo^2^ Em ambas as técnicas, o brilho de cada *voxel* é definido pela distribuição dessas propriedades de luz. Para imagens em volume, as diferenças de luz emitidas para os *voxels* são baixas. Em contraste, a cinematográfica utiliza um modelo de iluminação mais complexo, com o efeito da iluminação para outros *voxels* e reflexões subsequentes. Além disso, o sistema calcula o bloqueio da luz por partes do corpo para as outras estruturas e produz a sombra nas imagens, resultando em um efeito mais realista de profundidade.^1,2^ É fato que há uma demanda de potência computacional mais alta para a técnica cinematográfica, devido ao modelo de iluminação mais complexo. Assim, a exibição em tempo real e a manipulação tridimensional − por exemplo, girar a imagem −, são atualmente interrompidas por processos repetitivos de cálculo. A renderização da imagem final precisa de alguns segundos, variando de 5 a 30 segundos, dependendo da qualidade da imagem resultante.^1,2^

As técnicas de processamento e segmentação com reconstrução tridimensional das imagens médicas permitem a criação de modelos anatômicos reais baseados em dados de pacientes. Assim, qualquer imagem tridimensional é um modelo autêntico, real da doença a ser tratada.^4,6^ Para tal, é necessária uma TC com cortes finos, abaixo de 3mm, para gerar imagens adequadas. Os programas de desenho assistido por computador necessitam de muitas imagens (acima de 200 cortes em arquivos DICOM), para segmentação e produção de uma malha de polígonos tridimensional com qualidade.^1,4^ Exames com menos de 100 imagens ou com cortes ao redor de 10mm geram muita interferência, e as reconstruções ficam grosseiras e com uma qualidade ruim.^1,2,4^

Os modelos tridimensionais reconstruídos, virtuais ou impressos, possibilitam a reprodução de estruturas anatômicas sofisticadas que podem ser utilizadas para facilitar o estudo anatômico, o planejamento cirúrgico e o desenvolvimento de novas técnicas ou dispositivos. Estamos diante de uma revolução.^4-7^

### Oportunidades potenciais

Os três grandes campos para aplicação da tecnologia tridimensional em medicina são o ensino médico, tanto para graduação como residência; o planejamento e simulação em cirurgia; e a educação para o paciente.^4-6^

### Ensino de anatomia

A imagem médica, no entanto, tornou-se ferramenta cada vez mais importante no ensino de anatomia. A tecnologia tridimensional tem o potencial de melhorar ainda mais a anatomia virtual e acrescenta valor aditivo com a inclusão de imagens mais realistas, baseadas em exames de pacientes.^4,6,8^ Isso não só da anatomia normal como também das mais diversas doenças. Os alunos poderão interagir com a anatomia de vários pacientes e diversos tipos de doença a qualquer momento, por uma estação de trabalho de tecnologia tridimensional.^6,8^

### Educação paciente

A tecnologia tridimensional pode melhorar a comunicação com o paciente, pela qualidade das imagens. “Uma imagem vale mais que mil palavras”. Por meio das imagens tridimensionais, médicos e cirurgiões podem ilustrar para os pacientes as características de sua doença e demonstrar os possíveis tratamentos, muito diferente das imagens planas e transversais tradicionais em escala de cinza, nas quais a interpretação é raramente intuitiva para os pacientes.^4,6,8^ Além disso, as imagens geradas pela tecnologia tridimensional são fidedignas ao órgão real e à doença, sendo únicas e individualizadas. Em comparação com as imagens médicas tradicionais, a tecnologia tridimensional pode melhorar a compreensão e o envolvimento do paciente em sua doença e plano de tratamento.^3,7^

### Planejamento cirúrgico

Outro benefício é o planejamento pré-operatório e as simulações antes da intervenção. O planejamento do tratamento baseado em imagens e modelos produzidos pela tecnologia tridimensional é uma revolução no campo operatório.^3-5,7^ Cada vez mais as especialidades adotam e implementam esse recurso como parte da sua rotina.^5,7^

O planejamento cirúrgico é realizado pela integração mental de múltiplas imagens e depende da experiência do cirurgião. Médicos recém-formados apresentam dificuldades em compreender as alterações anatômicas em três dimensões somente pela avaliação de uma tomografia comum.^4-8^ Já a tecnologia tridimensional gera uma representação realista das estruturas de interesse com uma única imagem. Além disso, a manipulação das imagens permite a visualização de detalhes anatômicos por diferentes perspectivas. A representação mais realista das imagens, do que as obtidas tradicionalmente (transversais ou planas), deve ser mais próxima do que os médicos veem nas cirurgias e procedimentos − possivelmente com menor impacto de descobertas acidentais inesperadas e variações anatômicas.^4,6,7^ Em estudo sobre tumores da pelve, os autores destacaram o potencial da reconstrução cinematográfica para diagnóstico de estabelecer os limites dos tumores, o planejamento pré-operatório e a comunicação com os pacientes.^9^ Em outro estudo, realizado com 18 cirurgiões para comparar a renderização cinematográfica e TC, para compreensão da anatomia cirúrgica tomografia de 40 pacientes, a visualização com a primeira permitiu uma compreensão mais correta e rápida da anatomia cirúrgica em comparação com a tomografia convencional, independentemente do nível de experiência do cirurgião.^10^

### Limitações e estratégias alternativas

Os desafios atuais da tecnologia tridimensional incluem qualidade da imagem de origem, custo, tempo e estrutura.^2,3,5,7^ O investimento em um laboratório anatômico tridimensional requer equipamentos, programas de informática e pessoal de suporte capacitado.^1-3^

Esta nova técnica tem o potencial de criar um novo paradigma na visualização anatômica virtual. Novos padrões das imagens nos laudos médicos devem levar em consideração as alterações importantes detectadas nos exames de imagem, representadas por reconstruções tridimensionais por volume ou cinematográficas. Não faz mais sentido disponibilizar 200 ou 300 imagens tomográficas convencionais ao invés de algumas reconstruções bem feitas do órgão acometido. Devemos considerar a diferença entre custo, armazenamento e qualidade da imagem tridimensional em relação às imagens axiais tradicionais da tomografia convencional.

Novos estudos são necessários para determinar a possível utilidade diagnóstica de exames com reconstruções cinematográficas em comparação com imagens convencionais ou reconstruções em volume.

## CONCLUSÃO

Técnicas tridimensionais de reconstrução, como o volume, desempenham papel cada vez mais importante em diferentes áreas médicas. A renderização cinematográfica é uma nova técnica de reconstrução tridimensional. Por meio dela, temos uma representação realística da anatomia, com melhor forma e percepção de profundidade. Há um potencial amplo de aplicações, como ensino médico, planejamento cirúrgico e também para a percepção da doença pelo paciente.
